# Visualising dementia in news media: reinforcing ageing, dependency and stigma

**DOI:** 10.1093/ageing/afaf305

**Published:** 2025-10-21

**Authors:** Sanna-Maria Nurmi, Arja Halkoaho, Johanna Krüger, Henna Nikumaa, Anna Mäki-Petäjä-Leinonen, Kasper Katisko, Eino Solje

**Affiliations:** Institute of Clinical Medicine, Neurology, University of Eastern Finland, School of Medicine, Kuopio, Finland; Health and Well-Being, Turku University of Applied Sciences, Master School, Turku, Finland; Tampere University of Applied Sciences, Tampere, Finland; Neurocenter, Neurology, Oulu University Hospital, Oulu, Finland; Research Unit of Clinical Medicine, Neurology, University of Oulu, Oulu, Finland; Oulu University Hospital, MRC, Oulu, Finland; Faculty of Social Sciences and Business Studies, Law School, University of Eastern Finland, Joensuu, Finland; Faculty of Social Sciences and Business Studies, Law School, University of Eastern Finland, Joensuu, Finland; Institute of Clinical Medicine, Neurology, University of Eastern Finland, School of Medicine, Kuopio, Finland; Institute of Clinical Medicine, Neurology, University of Eastern Finland, School of Medicine, Kuopio, Finland; Neuro Center—Neurology, Kuopio University Hospital, Kuopio 70029, Finland

**Keywords:** dementia, media, a visual thematic analysis, stigma, media representation, qualitative research, older people

## Abstract

**Background:**

Media coverage shapes public perceptions of dementia. However, visual representation remains unexplored. This study examines how dementia is portrayed in Finnish news media.

**Methods:**

Visual thematic analysis with quantification was conducted on 255 images published between 2018 and 2021 in Finland’s four most-read national tabloids.

**Results:**

Media portrayals were predominantly negative and stereotypical, emphasising ageing, dependency and anxiety. People with dementia were mainly depicted as older, with grey hair, wrinkled hands and as individuals in need of assistance. Representations lacked diversity in terms of gender, ethnicity and experiences of dementia in daily life.

**Discussion:**

Newspaper images often reinforce stigmatising views of dementia by depicting dementia exclusively as ageing and dependency. Media should use more diverse and accurate visuals to reduce stigma and promote a more inclusive and realistic understanding of dementia.

## Key Points

- Visual representations of dementia in Finnish tabloid newspapers are predominantly negative and stereotypical.
- People with dementia are often portrayed as older, passive and dependent, which reinforces societal stigma.
- These findings underscore the importance of more balanced and inclusive imagery to promote a dementia-friendly public discourse.
- Healthcare professionals should support accurate and inclusive media portrayals to help reduce dementia-related stigma.
- The media lacks diverse images of people with dementia in terms of age, gender, ethnicity and lived experiences.

## Background

An estimated 55 million people worldwide are living with dementia, and this number is expected to double by 2030 and triple by 2050 [1]. While dementia predominantly affects older adults, ~10% of the cases are diagnosed before the age of 65, categorised as early-onset dementia (EOD) [2]. Dementia impacts not only the individuals diagnosed with dementia, but also imposes significant emotional, social and caregiving burdens on their families and loved ones [3–5].

Dementia is frequently associated with negative emotions, fear and stigma [6, 7], further complicating the experience for affected individuals and their families [5]. The 2024 World Alzheimer Report found that 88% of people with dementia have faced discrimination [8]. Stigma is a social process in which individuals are devalued, discredited and excluded based on characteristics such as illness, disability or identity [9]. Erving Goffman (1963) defined stigma as an attribute that reduces a person ‘from a whole and usual person to a tainted, discounted one’ [10]. It is maintained through stereotypes (generalised group-based beliefs), prejudice (negative emotions) and discrimination (unjust treatment) [9]. Stereotypes can serve as the basis for prejudice, which may lead to discriminatory behaviours such as social exclusion or unequal access to care [11, 12].

Media coverage plays a crucial role in shaping societal perceptions and understanding of dementia, serving as an important source of information for the general public and policymakers [6, 7, 13–17]. Stereotypes about people with dementia are conveyed through language and imagery [8]. Media often portray dementia in terms of ageing, cognitive decline and dependency, typically linked to Alzheimer’s disease and framed in biomedical or catastrophic terms [7, 13, 14, 18]. News frequently uses fear-inducing metaphors such as ‘tsunami’ or ‘worse than death,’ presenting people with dementia as passive or tragic victims [18–20]. Coverage emphasises medical treatments and scientific advances, giving less attention to prevention, coping or daily life of individuals with dementia [13, 18, 21–23]. The condition is often framed as a societal ‘burden’, with focus on costs and negative impacts [18, 24]. Visual representations similarly stress deterioration and loss, using symbolic imagery like wrinkled hands, blurred faces or fragmented brains [5, 25]. Visual media holds a powerful role in dementia disclosure [5] as images evoke immediate emotional responses, reinforce or challenge stereotypes and influence public understanding even more effectively than text [5, 7].

Media can influence dementia-related stigma across all defined levels of stigma: public, self and structural. Its most pronounced impact is on public stigma, which may serve as a basis for the development of self- and structural stigma [9, 11, 12, 26]. Public stigma refers to the negative community attitudes, beliefs and discriminatory actions directed toward individuals living with conditions [9, 11, 26]. Self-stigma often arises in response to public stigma, when individuals adopt negative societal beliefs about themselves [11, 26]. This can lead to feelings of shame, social withdrawal and reluctance to seek help [26]. Structural stigma becomes evident when media narratives influence political and institutional practices, such as resource distribution and service accessibility [9, 11].

News media shapes public perception through four key pathways grounded in established theories: agenda setting, framing, priming and persuasion [27]. Agenda setting directs attention to selected issues by giving them high visibility, shaping what audiences see as important [27]. Framing is the selection and presentation of information from a particular perspective, influencing how audiences understand and interpret issues being covered [27, 28]. Priming refers to how repeated exposure to specific content can unconsciously shape people’s perceptions, judgements and behaviours [27, 29]. Persuasion uses tactics such as expert quotations, statistics and images to enhance credibility and influence opinion, with repetition and consistency amplifying these effects [27]. Media platforms also enable other actors, including commercial interests, to steer public discourse. Together, these mechanisms can reinforce dementia-related stigma or promote more accurate and nuanced understandings, influencing public attitudes, policy priorities and the quality of care and inclusion for people living with dementia [5, 13].

Stigma surrounding dementia has harmful and far-reaching consequences [9, 10]. It can delay diagnosis, limit access to care and support, and contribute to social isolation. As a chronic psychological stressor, it may impair emotional, cognitive, behavioural and physiological functioning, reducing quality of life [9, 26, 30]. Stigma also creates barriers to the realisation of the legal rights, including access to healthcare, social benefits and equal participation in society [9]. In this way, stigma not only shapes public perceptions of dementia but also profoundly influences how those affected experience the condition and manage their everyday lives [5, 13].

Increasing research attention has focused on the representations of dementia in media narratives [5, 31]. While prior research has examined dementia in the media, most studies have focused on textual analysis of news stories, policy documents or popular culture, consistently showing that dementia is framed through biomedical, catastrophic and dependency-oriented narratives that reinforce stigma and narrow public understanding [7, 13, 18, 31]. A smaller body of work has considered visual portrayals, but these studies have typically been qualitative, anecdotal or embedded within broader textual analyses, leaving the visual dimension underexplored. To our knowledge, no study has conducted a systematic, large-scale visual thematic analysis of dementia imagery in news media. This study addresses that gap by analysing 255 images published in Finland’s four most-read national tabloids between 2018 and 2021. It contributes methodologically by demonstrating the value of systematic visual analysis, theoretically by linking imagery to stigma theory to show how portrayals reinforce ageing and dependency stereotypes and marginalise younger and minority groups, and practically by providing evidence-based recommendations for journalists, editors and public health communicators. Situated in a Nordic welfare state, the findings also offer insights relevant to societies beyond Finland.

## Methods

### Design

This study employed a qualitative research design to explore visual representations of dementia in Finnish media. The data consisted of 255 images published in Finland’s four most-read national tabloid newspapers between 2018 and 2021. To analyse these data, the study used visual thematic analysis [32] with quantification, chosen for its ability to identify and categorise recurring visual patterns into meaningful themes that reflect common representations of dementia in the media [32–34].

The analysis focused on manifest content, referring to the clear and explicit visual elements present, rather than underlying symbolic or metaphorical meanings that require more in-depth interpretation [32]. This approach was considered suitable because of the large dataset and its ability to identify the most immediately conveyed dominant and recurring visual messages. These consistent patterns reveal how certain perspectives and messages are consistently associated with dementia in the media, potentially shaping public understanding of the condition [32–34].

### Data collection and screening

We sampled images from four of Finland’s national newspapers, which provided both regional and national perspectives on the visual representation of dementia. The image search was conducted in June 2023 using the National Library of Finland’s search database. The University of Eastern Finland is part of the Tutkain agreement between the National Library of Finland and Kopiosto (the Finnish copyright management organisation), granting researchers access to selected digitised newspapers and journals published by the end of 2021 [35].

The search was limited to articles published between 2018 and 2021. The Finnish search terms used were ‘Muistisairaus’ (translation: Memory disease), ‘Alzheimerin tauti’ (translation: Alzheimer’s Disease), ‘Otsalohkodementia’ (translation: Frontotemporal dementia), and ‘Dementia’ (translation: Dementia). Articles were included if they focused on neurocognitive dementias and excluded if they were feature articles, opinion pieces, reviews of television programmes, films or books, advertisements or listings of television programmes. A total of 2086 records were screened, resulting in the selection of 182 articles. From these articles, 255 images were extracted for further analysis (Table 1).

**Table 1:** Description of the selected data.

Year	Number of images	Number of articles
2018	51	44
2019	75	51
2020	67	43
2021	62	44
**Total**	**255**	**182**

### Data analysis

First, the images were coded using coding framework (Table 2) to provide a general overview of the data. Each image was coded according to the framework by the first author, with ambiguous cases discussed with the second author to reach consensus. After coding, category frequencies and percentages were calculated based on the analytical framework.

**Table 2:** The coding framework.

Category	Description
Publication year	2018–2021
Type of image	Classification of image type (e.g. photograph, drawing, infographic, cartoon, brain scan).
Main subject	Primary focus of the image [individual(s), objects, landscapes, buildings, food and drink, other].
Gender of the person (if present)	Gender representation in the image (male, female, mixed, ambiguous, unidentified, not applicable).
Ages of the person (if present)	Age group represented in the image (child: 0–16, young adult: 17–30, middle-aged adult: 31–60, older adult: 60+, unidentified, not applicable).
Race of the person (if present)	Racial identity of the person depicted (White/Caucasian, Black/African, Asian, Hispanic/Latino, Middle East and North Africa Mixed race, unclear/unidentified).
Stock photo (if a person is present)	Whether the image appears to be a stock photo (Yes/No/Unidentified).
Environment	Setting depicted in the image (healthcare facility, public space, outdoor/nature, unidentified, not applicable).
Image alignment with article content	Assessment of whether the image corresponds with the article’s content (Yes/No).
Focus on Early-Onset	Indication of whether the article focuses on EOD (Identified from the article’s content) (Yes/No).
	

A visual thematic analysis of the images was carried out following Braun and Clarke’s six-step framework [32]. This framework provides a clear and systematic process for analysing qualitative visual data [33, 34]. It was chosen because it is widely used, flexible and well-suited to analysing diverse visual material, enabling both detailed examination and quantification of recurring themes. The structured process ensures a rigorous and transparent approach [34] and makes the data more manageable, facilitating the identification of clear themes within a large and diverse visual dataset [32–34]. The phases of the analysis are not strictly linear, instead, the process is iterative, often involving returning to earlier stages to refine codes and themes to make sure they accurately reflect the data [32].

First, the research team familiarised themselves with the images (Phase 1). Three team members from different professional backgrounds independently reviewed the data, then generated initial codes from key visual elements (Phase 2). For example, fresh vegetables and fruits were coded as ‘healthy diet’ and pills spilling from a bottle as ‘medication’. Through collaborative discussion, a structured codebook was developed, and codes were grouped into sub-themes and broader thematic categories to capture recurring visual patterns (Phase 3). These themes were iteratively reviewed and refined in discussions to ensure coherence and accuracy (Phase 4). Themes were further reviewed, defined and finally named (Phase 5). Finally, the findings were documented in a written report (Phase 6). Due to copyright restrictions, the images analysed cannot be reproduced.

## Results

### Description of the data

Most images (90%, 227/255) were photographs, followed by drawings (9%, 23/255) and brain scans (2%, 5/255). Most images depicted people (73%, 187/255), with objects (17%, 44/255), food and drink (7%, 17/255) and buildings or rooms (2%, 5/255) were less common. Infographics were rare, appearing in only 1% (2/255) of the images.

Amongst images where the environment could be identified (139/255), healthcare settings were most common (36%, 50/139), followed by home or home-like settings (35%, 48/139), outdoor or natural settings (20%, 28/139) and public spaces (9%, 13/139).

Ageing was strongly associated with dementia in the visual material. In images where the age of the person could be identified (136/255), 65% (89/136) depicted older adults (aged 60 and over), 27% (37/136) depicted middle-aged adults (aged 31–60) and 7% (9/136) showed mixed age groups. Children (aged 0–16) were rarely depicted, appearing in only 1% (1/136) of the images. Physical signs of ageing, including wrinkles, grey hair, frailty and physical incapacity, were commonly emphasised.

In images where gender could be identified (139/255), women were more frequently represented, appearing in 47% (65/139) than men (26%, 36/139). Mixed-sex groups appeared in 27% (38/139) of the images. All individuals depicted were White, indicating a lack of racial diversity. Of the images depicting people, 72% were stock images (135/187).

Overall, 64% (164/255) of the images were consistent with the content of the accompanying article, while 36% (91/255) were unrelated or potentially misleading. For example, an article discussing EOD was illustrated with an image of an older person’s hands assembling a jigsaw puzzle, and an article on prevention featured an older person leaning on their hands with an anxious expression. A total of 13% (33/255) of the images focused specifically on EOD, which aligns broadly with global prevalence estimates. However, these images were clustered within a small number of personal stories, leaving EOD relatively absent from the wider coverage.

### Results of the visual thematic analysis

Visual thematic analysis of news media images identified five key themes: (i) Preventing Cognitive Disorders through Lifestyle Choices, (ii) Risk Factors for Diseases Causing Dementia, (iii) Clinical and Scientific Perspectives on Dementia, (iv) Experiences of Dementia in Daily Life and (v) Social Relationships (Table 3).

**Table 3:** Results of the visual thematic analysis.

Main-theme	Sub-theme
**Preventing cognitive disorders through Lifestyle Choices** (*n* = 46)	A healthy diet (*n* = 20)
	Physical activity (*n* = 18)
	Cognitive exercise (*n* = 8)
**Risk Factors for Diseases Causing Dementia** (*n* = 28)	Lifestyle Factors (*n* = 6)
	Psychological factors (*n* = 7)
	Biological Factors (*n* = 15)
**Clinical and Scientific Perspectives on Dementia** (*n* = 41)	Pharmacological Treatments and Vaccines (*n* = 19)
	Scientific Research and Diagnostic Practices (*n* = 14)
	Brain Structure and Function (*n* = 8)
**Experiences of Dementia in Daily Life** (*n* = 106)	Memories in Photographs (*n* = 12)
	Symbolic Representations of Brain Atrophy and Memory Loss (*n* = 19)
	Functional Decline in Daily life (*n* = 35)
	Emotional and Psychological Burden of Dementia (*n* = 40)
**The Social aspects of Dementia** (*n* = 34)	Shared Moments and Close Relationships (*n* = 16)
	Social Support and Care (*n* = 18)
	

The main theme, ‘Preventing Cognitive Disorders Through Lifestyle Choices’ (*n* = 46), consisted of three sub-themes, each illustrating different strategies for dementia prevention. ‘The healthy diet sub-theme’ (*n* = 20) featured images of nutrient-rich foods, including fruits, vegetables, nuts, seafood and berries, emphasising the potential protective effects of a balanced diet against cognitive decline. These foods were often depicted in bright colours and aesthetically arranged. ‘The physical activity sub-theme’ (*n* = 18) depicted older adults engaging in exercise, including aerobic activities, flexibility training, dancing, walking, cycling and dog walking. Individuals in these images appeared happy, energetic and healthy, often exercising in groups. These images conveyed a visual message about the role of physical activity in maintaining cognitive health and reducing the risk of dementia. ‘The cognitive exercise sub-theme’ (*n* = 8) included images of older adults engaged in mentally stimulating activities such as playing chess, solving puzzles and playing musical instruments. These images emphasised the importance of cognitive engagement in dementia prevention. Although faces were often not visible, when shown, individuals appeared happy, active and healthy.

Another major theme identified was ‘Risk Factors for Diseases Causing Dementia’ (*n* = 28), which consisted of three sub-themes illustrating different risk factors. ‘The Lifestyle factor’s sub-theme’ (*n* = 6) represented behaviours such as alcohol consumption, unhealthy diet and smoking. For example, alcohol consumption was illustrated by an image of a silhouette holding a bottle with puzzle pieces falling from the head. ‘The Psychological Factors sub-theme’ (*n* = 7) focused on stress, negative emotions and sleep disturbances. For instance, stress was portrayed through images of individuals with strained expressions, such as a woman with illustrated smoke emerging from her ears and a fatigued businessman holding his forehead. Sleep disturbances were depicted with images of people anxiously staring at a clock or pulling their hair in frustration. ‘The biological Factors sub-theme’ (*n* = 15) included images related to high cholesterol, infections, high blood pressure and obesity. For example, infections were represented by images of a woman displaying herpes simplex lesions and another woman blowing her nose into a handkerchief.

‘Clinical and Scientific Perspectives on Dementia’ (*n* = 41) theme encompassed three sub-themes, each focusing on different aspects of the medical and scientific approach to dementia, including ongoing research efforts, treatment options and diagnostic processes. ‘The Pharmacological Treatments and Vaccines sub-theme’ (*n* = 19) included images of pills, pill bottles, people taking medication and healthcare professionals giving injections to patients. These images often depicted older adults taking medication or wrinkled hands holding pills or bottles. For example, one image showed a brain-shaped piggy bank into which blue and red pills were dropped. ‘The sub-theme of Scientific Research and Diagnostic Practices’ (*n* = 14) included images of healthcare professionals, such as doctors and researchers, engaged in diagnostic and research activities. These images depicted healthcare professionals standing next to imaging equipment, reviewing brain scans or conducting laboratory analyses, providing a visual insight into the diagnostic processes and scientific research related to dementia. ‘The sub-theme of Brain Structure and Function’ (*n* = 8) focused on the presentation of brain anatomy and physiology. The images often depicted the brain as a distinct anatomical entity, highlighting its structural complexity. Examples included infographics illustrating brain functions, images of doctors or researchers handling brain models, and brain images highlighting specific brain regions or their functions.

The theme, ‘Experiences of Dementia in Daily Life’ (*n* = 106), consisted of four sub-themes, each illustrating the impact of dementia on individuals living with the condition and their loved ones. The first sub-theme ‘Memories in Photographs’ (*n* = 12) featured images of older adults, alone or with loved ones, looking at old photographs. The images, often black and white, depicted significant life events such as weddings, family moments, military service or scenes from their youth. Photographs were a recurring visual element associated with memories, personal history and reminiscence. The second sub-theme, ‘Symbolic Representations of Brain Atrophy and Memory loss’ (*n* = 19) was illustrated by a variety of symbolic images depicting cognitive decline and memory loss. Several images showed a jigsaw puzzle in the shape of a human head silhouette, with missing pieces in the area where the brain would be. Only the puzzle maker’s chest was visible, leaving their identity unknown. There were also images of a human head silhouette cut out of paper, with small pieces blowing away in the wind. In some images, a human brain was drawn on paper and a person was erasing parts of it with an eraser. One picture showed a tree in the shape of a human head, with half its leaves missing and bare branches. Another showed portraits hanging on a wall with the faces erased, leaving only a blurred silhouette. In one picture, pieces of ice were floating in the water, arranged to resemble the silhouette of a human face in profile. Some of the pieces of ice had drifted away, breaking the silhouette. The third sub-theme ‘Functional decline in daily life’ (*n* = 35), which illustrated the progressive decline in both physical and cognitive functioning associated with dementia. The images depicted the impact of these impairments on daily activities, highlighting challenges in performing routine tasks and managing personal belongings. The depictions ranged from mild difficulties such as forgetfulness and disorientation to severe dependency requiring full-time care in a nursing home. Common images included misplaced objects (e.g. shoes in the dishwasher and hidden notes or piles of banknotes scattered around the living room), illustrating memory lapses. Other images showed older people experiencing cognitive decline—staring at their shoes in confusion, hugging a soft toy for comfort, wandering down a street with a forlorn expression on their face, or receiving help with personal care such as washing or nappy changing in a nursing home. Physical frailty was highlighted in images of people relying on canes or walkers or lying passively in a nursing home bed, emphasising their dependence on others. Assisted living solutions were also depicted, illustrating support systems to help individuals with daily tasks. Behavioural symptoms were represented, e.g. by an image of an older man looking angry and shaking his fist. The fourth sub-theme, ‘Emotional and Psychological Burden of Dementia’ (*n* = 40), was characterised by images of people showing emotional distress, often with sad expressions, heads bowed or resting on their hands. Some images showed blurred or indistinct faces, shadows resting on hands or people looking at their reflections in mirrors with sad expressions. Others showed people gazing into the distance with pensive expressions. In addition, many images showed people alone. Some showed a person, photographed from behind, walking alone. This sub-theme included a higher number of people of working age compared to other categories.

‘The Social aspects of Dementia’ (*n* = 34) theme encompassed two sub-themes and illustrated the crucial role of social connections in the well-being of individuals with dementia, as well as the impact of the condition on their family members and loved ones. The sub-theme of ‘Shared Moments and Close Relationships’ (*n* = 16) captured the role of meaningful social interactions in fostering emotional well-being. The images depicted shared moments between individuals with dementia and their loved ones, highlighting the warmth and joy found in family connections. Common depictions included couples, families and multiple generations engaging in everyday activities—sitting closely, looking at photographs, holding hands or hugging. Expressions in these images conveyed happiness and emotional closeness. The sub-theme ‘Social Support and Care’ (*n* = 18) emphasised the role of close relationships in providing both emotional and practical support to individuals with dementia. The images depicted caregivers assisting with daily activities, such as preparing meals, helping with dressing and offering physical reassurance through touch. Many images portrayed caregivers holding hands, comforting the person or simply being present.

## Discussion

Our study provides the systematic, large-scale visual thematic analysis of dementia imagery in national news media over several years in the Finnish context, addressing a dimension of media representation that has been largely overlooked in prior research. These findings reveal that the media often portrays dementia in a negative and stereotypical manner, reinforcing societal stigma. Dementia is predominantly depicted as a condition of ageing, anxiety and dependency, with limited attention paid to the possibility of living a meaningful and fulfilling life after diagnosis. In addition, media images lack diversity in terms of age, gender, background and experiences of dementia in daily life, failing to reflect the clinical diversity of the patient population.

This stereotypical portrayal is consistent with previous studies, which have shown that dementia is frequently framed through narratives of ageing, and dependency, often associated with Alzheimer’s disease and biomedical or catastrophic perspectives [7, 13, 14]. Such portrayal reinforce stigma by emphasising loss and suffering and depicting individuals with dementia as passive victims rather than cognizing their individuality and agency [5, 22, 25, 36, 37]. Media representations shape public attitudes, influence individuals´, families´ and healthcare systems´ responses and ultimately affect the quality of care received by the individuals with dementia [7, 13, 14, 36].

In line with previous findings [7, 13, 14], our results indicate that people with dementia are primarily depicted as older, reinforcing the misconception that dementia is simply a natural consequence of ageing rather than a neurological, biological disorder. According to previous research, this disparity is related to the fact that the rights of people with dementia are not upheld as equally as those of other disease and disability groups [38]. Although the proportion of images focusing on EOD matched prevalence estimates, they appeared mainly in a few feature articles. As a result, EOD remains invisible in broader coverage, reinforcing the misconception that dementia is exclusively a condition of old age. This limited portrayal can exacerbate stigma, delay diagnosis and increase social isolation for younger people living with dementia [22, 25, 37, 39, 40]. Mixing dementia with normal physiological ageing might have important implications for public opinion including scepticism toward early diagnosis and underinvestment in diagnostic infrastructure. For instance, one out of four Europeans do not have access even to MRI imaging as a part of diagnostic procedures and fewer than half have access to appropriate biomarkers [41] required for establishing diagnosis [42].

Our findings also reveal a lack of diversity in visual representation of dementia. Women were depicted more frequently than men and people from different ethnic minority backgrounds were completely absent. Prior research emphasises that dementia awareness and help-seeking behaviours are shaped by cultural values [39, 40] and a lack of diverse representation can further marginalise minority groups and undermine access to care [37, 40].

The portrayal of dementia predominantly emphasised emotional burden, anxiety, loss of function and dependency. While emotional burden is an important aspect of the experiences of dementia in daily life, the overrepresentation of sadness, loneliness and helplessness risks reinforcing misconceptions. It is important to note that symptoms such as depression and anxiety are not core clinical features of Alzheimer’s disease but may occur as comorbidities [43]. Although some images captured elements of the daily experiences of people living with dementia, the overall negative and stereotypical nature of the portrayals reinforces stigma and overlooks the complexity of the condition [40–42].

Dementia encompasses over 100 different diseases, and many individuals maintain independence and quality of life for extended periods following diagnosis. Previous studies have shown that people with dementia often report being relatively satisfied with their lives [44, 45], even more so than their family caregivers [46, 47]. Therefore, media should aim to portray individuals with dementia as active and engaged members of society, reflecting the reality of their lived experiences more accurately.

The media occasionally depict dementia in a positive light, particularly in contexts that align with ideals of an active lifestyle or close social relationships. Such portrayals, emphasising togetherness or dementia prevention through physical activity, often frame individuals with dementia as happy and successful only when supported by others, not independently. This reinforces the notion that people with dementia cannot experience well-being on their own. The WHO’s ‘active ageing’ paradigm promotes optimising health, participation and security to enhance quality of life in later years [48]. While it has shaped ageing policy and public discourse internationally, it has been criticised for privileging autonomy, productivity and physical fitness, thereby marginalising older adults who cannot meet these ideals due to illness or disability [49–51]. People with dementia are often excluded from this idealised image, as media rarely depict them as active participants, and the model’s emphasis on autonomy, productivity and decision-making conflicts with perceptions of cognitive decline [51]. Despite criticism [49, 51], media representations seem to continue reinforcing these values [51].

Furthermore, the depiction of lifestyle factors related to dementia prevention, such as diet and exercise, may promote healthy behaviours but risks oversimplifying dementia aetiology and emphasises individual responsibility. Furthermore, the strong presence of medical imagery, particularly medication use, may create a misleading impression that pharmacological treatments are curative, despite the current lack of such therapies. These images may reinforce and perpetuate hope and a sense of ‘cruel optimism’ [24].

A particularly interesting finding was that a significant portion of the images analysed (36%) did not align with the content of the accompanying articles. Image selection practices often prioritise emotional impact over informational accuracy, favouring emotionally charged, dramatic visuals. Practical constraints, such as reliance on image banks, also contribute to stereotypical depictions, as shown in previous studies [31]. It is important to acknowledge that living with dementia involves real challenges, and media representations should not overlook these difficulties. However, while it is appropriate for imagery to reflect the more demanding aspects of the condition, it is equally essential to maintain balance. Overemphasis on negative experiences risks reinforcing harmful stereotypes, whereas more nuanced portrayals can help foster a realistic and empathetic understanding of dementia.

Selecting images that emphasise social connection by showing people with dementia as active participants and engaging with others, conveys agency and inclusion [5, 8, 52]. Images should avoid depicting people with dementia solely as passive care recipients or victims. Including individuals from various stages of the condition and diverse backgrounds can deepen public understanding of dementia and its diverse manifestations, can broaden public understanding, challenge misconceptions, and counter the one-dimensional narrative that reduces dementia to ageing, loss and dependency. Showing everyday life, meaningful relationships and retained abilities presents individuals as more than their diagnosis, fostering empathy, reducing fear and social distance, and encouraging decisions that support the rights and well-being of those affected. Replacing metaphors of loss with those of transformation conveys adaptation, growth and new forms of connection. Seeing oneself or loved ones reflected in such images can further reduce stigma, with emotional responses shaping attitudes, behaviours, services and policy.

Over two-thirds of the images were stock photographs. While stock images can illustrate general concepts, they often rely on generic or stereotypical portrayals that fail to reflect the lived experiences of people with dementia. Overuse of such inauthentic images can reinforce oversimplified or misleading narratives [52]. Models are usually not living with the condition, settings are staged and the images may not directly relate to dementia. In contrast, authentic photographs created with people living with dementia and their families can provide richer, more realistic portrayals that challenge stigma and deepen understanding.

The media plays a crucial role in shaping societal perceptions of dementia and influencing how individuals with the condition perceive themselves. This study highlights the urgent need for more diverse, balanced and accurate visual representations, depicting individuals at different stages of dementia and from diverse backgrounds. By challenging stereotypes and promoting a more complete understanding of dementia, the media can contribute to stigma reduction and improve the well-being of individuals living with the condition. Showing people with dementia as active participants in everyday life and relationships fosters empathy, reduces fear and encourages social inclusion.

This study extends prior work in three important ways. First, it is the first to conduct a systematic, large-scale visual thematic analysis of dementia imagery in national news media, treating images as the primary object of analysis rather than a supplement to text. This methodological contribution highlights the importance of studying visual content systematically, given its strong emotional and persuasive impact on audiences. Second, by linking visual portrayals to stigma theory, our findings demonstrate how imagery reinforces ageing and dependency stereotypes, conflates dementia with ‘normal’ ageing, and omits younger and ethnically diverse groups. In doing so, the study shows how media representations not only reflect but also contribute to public, self and structural stigma. Third, by situating the analysis in a Nordic welfare state context, the findings provide comparative insights relevant for other societies with advanced elder care systems but persistent stigma, making them valuable for international debates.

### Strength and limitations

A key strength of this study is the multidisciplinary research team, which conducted the analysis collaboratively, ensuring a balanced and comprehensive interpretation of the data. Another significant strength is the large dataset of 255 images collected from national news articles published between 2018 and 2021, enabling the first systematic, longitudinal analysis of visual portrayals of dementia in Finnish media. By focusing exclusively on images, this study also adds a methodological innovation to the field, treating visuals as central rather than supplementary to textual analysis. Situating the findings within a Nordic welfare state context further strengthens their broader relevance, as the results are consistent with international research yet also provide insights from a society with advanced elder care systems but persistent stigma. Certain limitations should be acknowledged. First, the study analysed only Finnish media, which may limit generalisability. However, the alignment of our findings with previous research from other Western contexts suggests that that the observed patterns are not unique and may have broader relevance. Second, while the focus on visual material allowed for depth and rigour, a combined visual-textual analysis could provide additional insights into how images and language interact in shaping meaning. Finally, the exclusion of other visual formats such as video, animation and infographics may not fully capture the broader media landscape. These limitations nonetheless point to fruitful avenues for future research, which could build on our findings by comparing multiple countries, media types and multimodal representations to deepen understanding of how dementia is portrayed globally.

## Conclusions

This study is the first to systematically analyse visual portrayals of dementia in Finnish national news media over several years. The results of this study provide novel insights into how dementia is visually represented in the tabloid news media, revealing a stereotypical imagery that frames the condition primarily through ageing, negative emotions and dependency. Media portrayals overwhelmingly emphasise physical signs of ageing, such as grey hair and frailty, reinforcing narrow stereotypes and failing to capture the real-life complexity of dementia. By situating the findings within the Finnish welfare state context, the study offers insights relevant for international comparison, showing that stigma can persist even in societies with advanced health care systems.

The findings show a lack of diversity, with minimal representation of younger individuals or people from different ethnic backgrounds. Depictions frequently present individuals with dementia as passive and dependent, overlooking their capacity for autonomy, participation and meaningful engagement in society. Such portrayals can influence how dementia is understood by the public, potentially reinforcing misconceptions, contributing to stigma, and shaping attitudes, health-seeking behaviours and policy priorities.

Given the media’s powerful role in shaping public discourse and influencing societal attitudes, including political decisions, there is an urgent need to broaden the visual narratives surrounding dementia. The research-informed recommendations provide practical tools for journalists, editors and public health communicators to select imagery that is more diverse, accurate and empowering. In addition, more diverse, accurate and empowering portrayals are essential to challenge stigma, foster greater public understanding, and ultimately support the inclusion and well-being of people living with dementia and their families.
